# Topological phase transition and quantum spin Hall edge states of antimony few layers

**DOI:** 10.1038/srep33193

**Published:** 2016-09-14

**Authors:** Sung Hwan Kim, Kyung-Hwan Jin, Joonbum Park, Jun Sung Kim, Seung-Hoon Jhi, Han Woong Yeom

**Affiliations:** 1 Center for Artificial Low Dimensional Electronic Systems, Institute for Basic Science (IBS), Pohang 37673, Republic of Korea; 2 Department of Physics, Pohang University of Science and Technology (POSTECH), Pohang 37673, Republic of Korea

## Abstract

While two-dimensional (2D) topological insulators (TI’s) initiated the field of topological materials, only very few materials were discovered to date and the direct access to their quantum spin Hall edge states has been challenging due to material issues. Here, we introduce a new 2D TI material, Sb few layer films. Electronic structures of ultrathin Sb islands grown on Bi_2_Te_2_Se are investigated by scanning tunneling microscopy. The maps of local density of states clearly identify robust edge electronic states over the thickness of three bilayers in clear contrast to thinner islands. This indicates that topological edge states emerge through a 2D topological phase transition predicted between three and four bilayer films in recent theory. The non-trivial phase transition and edge states are confirmed for epitaxial films by extensive density-functional-theory calculations. This work provides an important material platform to exploit microscopic aspects of the quantum spin Hall phase and its quantum phase transition.

Topological insulators (TI’s) possess distinct properties, among which most important is the existence of spin helical Dirac fermions on their edges protected robustly. Such topological edge states (TES’s) have been fully demonstrated through experimental studies^1^,^2^,^3^,^4^,^5^,^6^ and grant attractive applications in transporting spin current for spintronic devices and in providing key elements of topological quantum computers^7^,^8^. Two-dimensional (2D) TI’s are particularly interesting in applications because of their merits in fabricating electronic or spintronic device structures with their intrinsically localized edge channels. Their TES’s represent two spin polarized channels, which correspond to quantum spin Hall (QSH) edge states. The 2D TI phase was established in HgTe/CdTe and InAs/GaSb quantum well structures through transport measurements^9^,^10^,^11^,^12^ and ultrathin films of a 3D TI Sb_2_Te_3_, Bi_2_Te_3_, and Bi_2_Se_3_ were suggested to fall into the 2D TI phase^13^,^14^,^15^,^16^,^17^,^18^,^19^,^20^. Edge channels of 2D TI’s were accessed microscopically for quantum well structures with scanning SQUID but with only micrometer resolution^21^,^22^. More recently, scanning tunneling microscopy/spectroscopy (STM/STS) studies^23^,^24^,^25^,^26^,^27^,^28^,^29^ claimed the truly microscopic and direct observation of a nanometer scale TES of a 2D TI for a Bi single bilayer (BL) film in which the QSH phase was predicted earlier^30^,^31^. However, these Bi single layer films were realized on substrates with strong interactions, which leaves large ambiguity in the topological nature of their edge states^26^,^31^,^32^,^33^,^34^,^35^,^36^.

In this work, we report the realization of the 2D QSH phase in another material based on epitaxially grown thin films and demonstrate its merit in the unambiguous microscopic observation of a TES. We note on the recent theoretical prediction of a series of topological quantum phase transitions in ultrathin Sb films, particularly that from trivial to the semi-metallic QSH phase between 3 and 4 BL thickness^37^. We investigate local electronic structures of ultrathin Sb films grown on Bi_2_Te_2_Se by STM/STS and *ab initio* calculations. The initial growth of Sb on Bi_2_Te_2_Se exhibits islands of various heights with well-ordered zigzag edges. Our spatially resolved *dI/dV* (STS) measurements clearly identify strong and robust edge electronic states for 4 and 5 BL films in contrast to 3 BL films. Our calculations clearly indicate the topological phase transition between 3 and 4 BL’s and the topologically non-trivial edge states for 4 BL’s both without and with the interaction of the substrate. A thin film based 2D TI with a well resolved TES is thus established.

## Results

### Sb film growth on Bi_2_Te_2_Se

**Figure 1:**
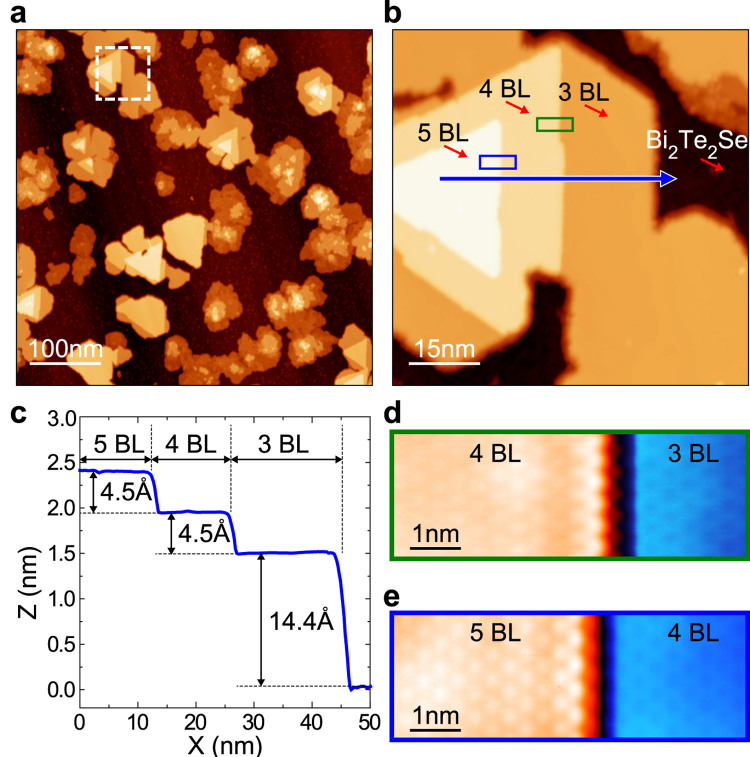
Sb film growth on Bi_2_Te_2_Se. (**a**) STM image for Sb films and islands on Bi_2_Te_2_Se (tunneling conditions of V_S_ = 1 V and I_T_ = 50 pA). Well ordered Sb islands appear over 2 BL thickness. (**b**) Close-up STM image for an ordered Sb island, indicated by the dashed rectangle in (**a**). (**c**) The height profile of the island along the blue arrow in (**b**). The single layer height is about 4.5 Å corresponding to one Sb(111) BL^38^,^42^. The step edge structures of (**d**) 4 and (**e**) 5 BL parts, the green and blue rectangles in (**b**), respectively. Zigzag chains of edge atoms are clearly resolved (V_S_ = +0.1 V, I_T_ = 1.5 nA).

Figure 1a presents topographic STM image of Bi_2_Te_2_Se where Sb islands of 1–5 BL heights are formed. At this growth condition, single BL islands are rare and the portions of 2, 3, 4, and 5 BL areas are about 18, 18, 8, and 2%, respectively. In particular, islands of 3, 4, and 5 BL heights have compact (Fig. 1b,c) and atomically well ordered structures as shown in Fig. 1d,e. The surface of 2 BL films is rather disordered with vacancies and clusters. These close-up images show unambiguously that the islands are (111) oriented with a lattice constant of about 4.0 Å, being consistent with the previous report^38^. Their well defined edges are perpendicular to the 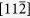 direction (parallel to 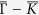 direction in the *k* space) and have the zigzag atomic structure.

### Local electronic structure of Sb(111) films on Bi_2_Te_2_Se

**Figure 2:**
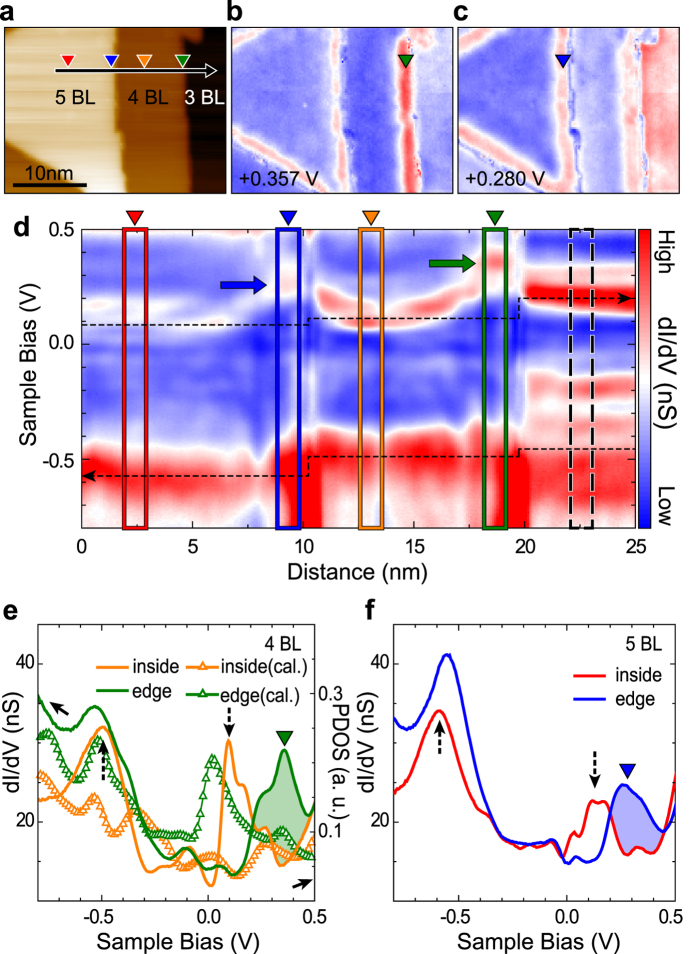
Local electronic structures and edge states of Sb 4 and 5 BL. (**a**) STM topography image (30 nm × 22.5 nm) on Sb island obtained simultaneously with the STS (*dI/dV*) spectra, which contains the edges of 4 and 5 BL films. The *dI/dV* maps at two particular energies for edge states; (**b**) +0.357 V and (**c**) +0.280 V. (**d**) The 2D plot of the *dI/dV* line scan measured along the black arrow indicated in (**a**), which crosses two zigzag edges. Averaged *dI/dV* curves taken from the red, blue, orange, and green rectangles in (**d**), representing the center and edge parts of 4 and 5 BL films, are shown in (**e**,**f**), respectively. In (**e**), the corresponding calculated LDOS’s taken from the band structure calculations of Fig. 4 are shown together (see Supplementary Fig. 5 for more details). Around +0.36 and +0.28 eV, the strongly enhanced edge states appear [the green and blue arrows in (**d**) and arrow heads in (**e**,**f**)].

In order to reveal electronic structures of ultrathin Sb films, we performed *dI/dV* measurements on Sb islands, for example, for an island with 3, 4, and 5 BL height films together (Fig. 2a). We take detailed *dI/dV* curves along a line crossing two neighboring edges (the arrow within Fig. 2a). The result of this scan is shown in Fig. 2d. The notable features are strong spectral intensities at about −0.5 and +0.1 ~ 0.25 eV in filled and empty states, respectively (horizontal dashed lines). These energies correspond well to the valence band edges of the substrate and the hybridized state between conduction bands of the substrate and the Sb film, respectively (Supplementary Fig. 1). The gradual downshift of these features evidences the charge transfer between the film and the substrate, which depends on the film thickness. The *n*-type doping of the Bi_2_Te_2_Se substrate was also observed for the Bi film growth^19^. Between −0.5 and +0.1 eV, 2D valence states of the Sb film appear as weak spectral features (more details found in Supplementary Fig. 1). Even within this line scan, one can notice the rather regular spatial modulation of these 2D electronic states of Sb films (and also for those at +0.1~0.25 eV) due to the quasi-particle interference induced by the presence of step edges (Supplementary Fig. 2). The interference pattern analysis, data not shown here, supports their origin in valence states of the Sb film.

In addition to the spectral features within flat parts of Sb films, one can easily notice that electronic states are substantially modulated on step edges (blue and green rectangles in Fig. 2d), in particular in empty states as indicated by arrows. As detailed in Fig. 2e,f, there exist unique electronic states on the edge sites at about +0.36 and +0.28 eV for 4 and 5 BL films, respectively. In the 2D lateral map of *dI/dV* intensities at these energies, one can clearly see that these electronic states are strongly confined along the edges (Fig. 2b,c) within a width of 2 nm (Supplementary Fig. 3). This edge state cannot be explained by the energy shift of the neighboring state at +0.1 ~ 0.25 eV within the interior of islands since those states do not merge into the edge state and no band bending toward the edge is noticed for all the other spectral features (Supplementary Fig. 4).

**Figure 3:**
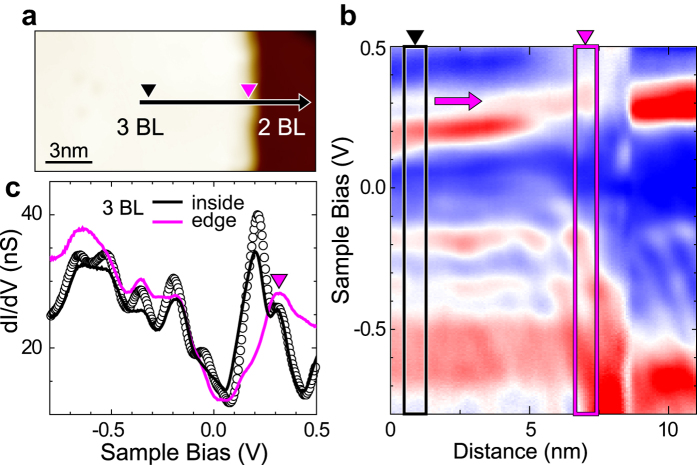
Local electronic states of Sb 3 BL. (**a**) STM topography image of a Sb island with its edge from a 3 BL film. (**b**) The 2D plot of the STS *dI/dV* line scan along the black arrow in (**a**). (**c**) Averaged *dI/dV* curves taken from the film and the edge (black and violet rectangles, respectively). The data in open circles are from a different 3 BL film shown in Fig. 2a (the dashed rectangle in Fig. 2d), exemplifying the consistency of the STS data over different islands and films.

We performed similar experiments for islands of 3 BL films on the same substrate. The edge-crossing *dI/dV* line scan for a 3 BL film is shown in Fig. 3. The 2D plot of this measurement is depicted on Fig. 3b. Even though the *dI/dV* spectra show rich spectroscopic features, the edge spectrum does not change drastically without a strong edge-localized feature (arrow in Fig. 3b,c). This indicates unambiguously that the edges of 3 BL and thicker films have distinct electronic properties (Supplementary Fig. 5) as discussed further below.

### Topological nature of ultrathin Sb films

**Figure 4:**
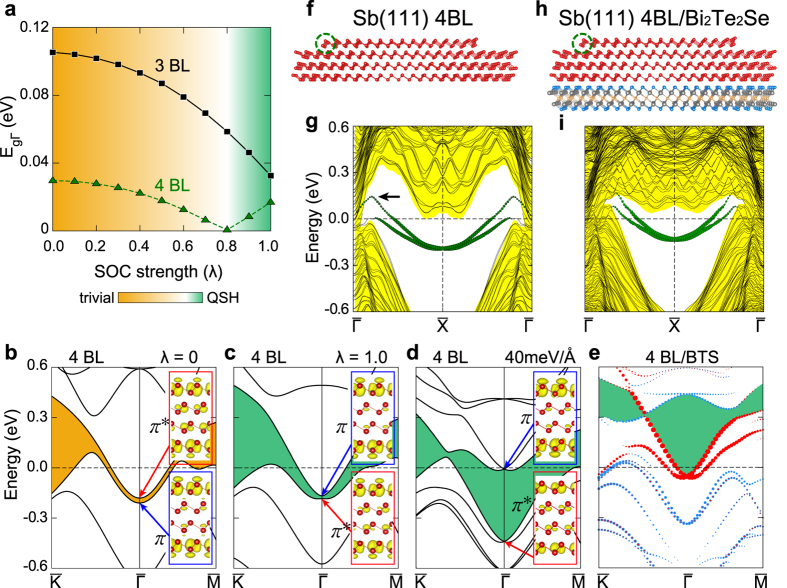
*Ab initio* calculations for Sb films and nanoribbon. (**a**) Calculated band gap at 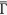 (E_*g*Γ_) with respect to the strength of spin-orbit coupling (SOC, λ) for 3 and 4 BL infinite Sb(111) films. The SOC strength (λ) is artificially set to partial fractions of the true value of SOC. The calculated band structures for a Sb 4 BL film are depicted for *λ* of (**b**) 0 and (**c**) 1 along the 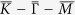 direction. The band inversion between *π* and *π*^∗^ states (insets) occurs after the reopening of the band gap over λ = 0.8. (**d**) The calculated band structure of Sb 4 BL when an electric field of 40 meV/Å is applied, The band inversion is maintained with this electric field. (**e**) Calculated band structure for Sb 4 BL films on Bi_2_Te_2_Se. The band structure is consistent with that in (**d**) except for an overall energy shift. The yellow and green shadows in (**b**,**c**–**e**) indicate the trivial and non-trivial band gaps, respectively. In (**e**), the red and blue dots represent the bands of the top and bottom layers of the Sb film, respectively (see Supplementary Fig. 6f for more details). The step edge structure of a 4 BL zigzag-edged Sb(111) nanoribbon (**f** ) without [(**h**) with] the substrate. The top layer has a nanoribbon geometry to generate step edges. The calculated band structure of a 4 BL film with step edges along the 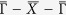 direction (**g**) without [(**i**) with] the substrate. The band in green dots are localized on the edge atoms [dashed circles in (**f** ) and (**h**)]. The edge states bands have two branches dispersing out from the conduction and valance bands and crossing at 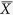 point to form a topologically non-trivial Dirac band.

According to the previous theoretical work for Sb films^37^, the bands of ultrathin Sb film undergo the band inversion between 3 and 4 BL’s, which indicates a topological phase transition. We detailed this result with our own calculation. We studied the band structure of free standing Sb films of various thickness as a function of the strength of the spin-orbit interaction. As shown in Fig. 4a, above the thickness of 4 BL’s, the band gap at the Brillouin zone center closes and reopens as the spin-orbit interaction is increased. This is directly related to the band inversion; the detailed band dispersion analysis shown in Fig. 4b,c indicates that the bonding and anti-bonding characters of the corresponding bands are switched at the full strength of the spin-orbit interaction. This topological phase transition changes the characteristics of edge states too. In Fig. 4g, we show the electronic band dispersion calculated for a 4 BL film. This film has the nanoribbon geometry for the top layer to feature step edges as shown in Fig. 4f. This geometry mimics well the island and edge structure of the experiment. At the step edge of the top layer of a 4 BL film, we can identify the edge state band. This band has a Dirac dispersion with its Dirac point at the 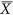 point, where the band gap is largest, and with its spin-split branches dispersing into valence and conduction bands separately at 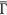. This clearly evidences the nontrivial TES character (Fig. 4g and Supplementary Fig. 5). Comparing with the STS spectra, we related the edge state peak of the experiment to the top of the edge state band (arrow in Fig. 4g) while there is some energy difference (about 0.12 eV) (Fig. 2e). The calculated LDOS for a TES has another strong peak at a lower energy near Fermi level. This comes from the degenerate TES bands around the K point, which are not easily probed by tunneling spectroscopy probing preferentially electronic states with small in-plane momenta. We prove further that the topological nature of the 4 BL film does not change upon the interaction with the substrate (Fig. 4h,i,e) and the epitaxial case is equivalent to the film under an electric field (Fig. 4d and Supplementary Fig. 6). For a thinner film, the edge state of 2 BL’s branches are spin-split but merge at 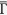 forming topologically trivial Rashba spin-split bands for both floating and epitaxial films (Supplementary Fig. 5).

## Discussion

The QSH edge state is claimed to be directly accessible in nanometer scale for Bi single BL films^25^,^26^. However, on the substrate like Bi_2_Te_3_ and Bi_2_Te_2_Se, the interaction with the substrate is strong enough to close the band gap and the topological nature of the edge state is not clear enough. On the other hand, the step edge state of the Bi surface layer^39^ was also claimed as the QSH edge state. However, the 2D TI phase of the Bi(111) surface layer cannot be justified and the observed edge state corresponds to the trivial Rashba spin-split state^32^. In the present case, the edge enhancement of the spectral feature is very much clear and the interfacial interaction is largely diminished for the top layer of 4 or 5 BL films (Supplementary Fig. 5). The robustness of the edge state over defects such as kinks is remarkable in the LDOS maps and spectra (Fig. 2 and Supplementary Fig. 2). Moreover, the absence of the edge state in thinner films corroborates the relationship of the edge state with the 2D topological phase transition. That is, while 2 and 3 BL films feature spin polarized Rashba-type edge states similar to a TES, it is not clearly observed in the experiment in clear contrast to the TES’s of 4 and 5 BL films. We believe that this reflects the susceptibility of a trivial edge state to defects and structural imperfections and, on the other hand, the robustness of a TES. These two facts, the great sensitivity to the film thickness and the total insensitivity to defects, manifest the non-trivial nature of the edge states observed here. The present observation is parallel to a largely different and very recent approach to the QSH edge state, the STM study of the step edge of a weak 3D TI, which corresponds to a stack of 2D TI layers^39^. This and the present work would uniquely make it possible to investigate microscopic details of a 2D QSH edge state. A controlled experiment with magnetic and nonmagnetic impurities would be helpful to reveal the topological nature of these states more clearly and would open a way to control them. In addition, the present system would make it possible to investigate the 2D topological quantum phase evolution from trivial to nontrivial 2D and 3D phases, which occur as a function of the film thickness.

## Methods

### STM/STS measurements

The STM/STS experiments were performed in ultrahigh vacuum better than 5 × 10^11^ Torr, using a commercial low temperature STM (Unisoku, Japan) at ~78 K. STM topographic images were obtained using the constant current mode. The STS spectra (*dI/dV* curves) were acquired using the lock-in technique with a bias-voltage modulation of 1 kHz at 10–30 mV_rms_ and a tunneling current of 500–800 pA.

### Sample growth

Bi_2_Te_2_Se single crystals were used as the substrate, which were grown using the self-flux method^26^,^32^. The single crystals were cleaved *in vacuo* at room temperature. Ultrathin Sb islands were grown by a thermal effusion cell at room temperature.

### Theory

*Ab initio* calculations were carried out in the plane-wave basis within the generalized gradient approximation for the exchange-correlation functional^40^,^41^. A cut-off energy of 400 eV was used for the plane-wave expansion and the *k*-points of 11 × 11 × 1 for the Brillouin zone sampling. In order to investigate edge states, we carried out calculations for a single BL nanoribbons of 15 Sb zigzag chains on top of flat and infinite 1 and 3 BL slabs. This reproduces step edges with the zigzag structure of 2 and 4 BL films. The Sb slabs and the nanoribbons were fully relaxed until the Helmann-Feynman forces were less than 0.01 eV/Å.

## Additional Information

**How to cite this article**: Kim, S. H. *et al*. Topological phase transition and quantum spin Hall edge states of antimony few layers. *Sci. Rep.* **6**, 33193; doi: 10.1038/srep33193 (2016).

## Supplementary Material

Supplementary Information
