# Supplementary material for: Topological phase transition and quantum spin Hall edge states of antimony few layers
Source: Sci Rep. 2016 Sep 14;6:33193. doi: 10.1038/srep33193 (PMC5021940; doi:10.1038/srep33193)
Supplement: Supplementary Information [file srep33193-s1.pdf]

Supplementary Information for

Topological phase transition and quantum spin Hall edge states of antimony few layers

Sung Hwan Kim,[1,2] Kyung-Hwan Jin,[2] Joonbum Park,[2] Jun Sung Kim,[2] Seung-Hoon Jhi,[2] and Han Woong Yeom[1,2]

[1]*Center for Artificial Low Dimensional Electronic Systems, Institute for Basic Science, Pohang 37673, Republic of Korea*
[2]*Department of Physics, Pohang University of Science and Technology, Pohang 37673, Republic of Korea*

A comparison between the calculated band structure and the STS spectrum for a Sb 4 BL film on Bi_2Te_2Se. The rich features of the *dI/dV* curve represent well the band structure. The states indicated by blue dashed lines originate mainly from the band edges of the substrate, which are located near +0.1 and -0.5 V. The other smaller features around -0.10, +0.15, and +0.27 eV (the red dashed lines) correspond to the states of Sb films, which have some degree of hybridization with those of Bi_2Te_2Se.

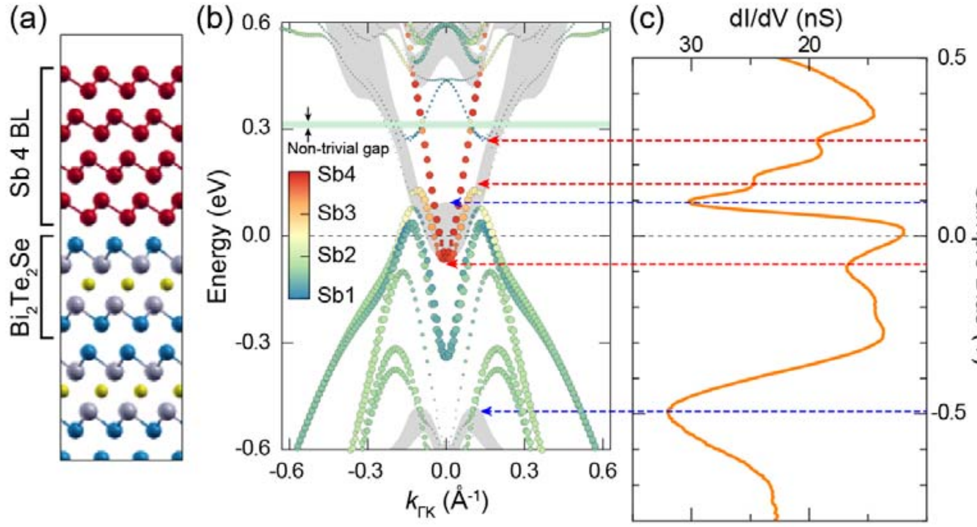

Supplementary Figure 1. The origins of Sb 4 BL's STS spectral features. (a) The atomic structure model for the *ab initio* calculation of Sb 4 BL's on Bi_2Te_2Se (BTS). The structure is simulated by a supercell with Sb 4 BL's on one surface of a slab of six quintuple layers BTS and a vacuum layer of 20 Å between the cells. During structural relaxation, the atoms of Sb film and BTS surface 3 layers are allowed to relax until the forces are smaller than 0.01 eV/Å and the van-der Waals interaction is considered. (b) The calculated band structure for the Sb 4 BL film on BTS. Colors indicate which Sb layer the states originate from. The electronic states of BTS are within the grey region. (c) The STS spectrum measured inside of Sb 4 BL's.

Local density of states (LDOS) measurements for the 4 and 5 BL Sb films. The edge localized electronic states are very pronounced with well defined energies at +0.36 and +0.28 eV [(g) and (f)], for the 4 and 5 BL Sb films, respectively. In the range from +0.008 to +0.16 eV, the quasi-particle interferences within the films are clearly observed, which originate from the electron scattering of the surface states of the film by step edges [(b)-(e)]. The edge states are very robust against the defects so that their LDOS are affected only marginally on defects such as kinks [(h) and (i), the arrows in (f) and (g)].

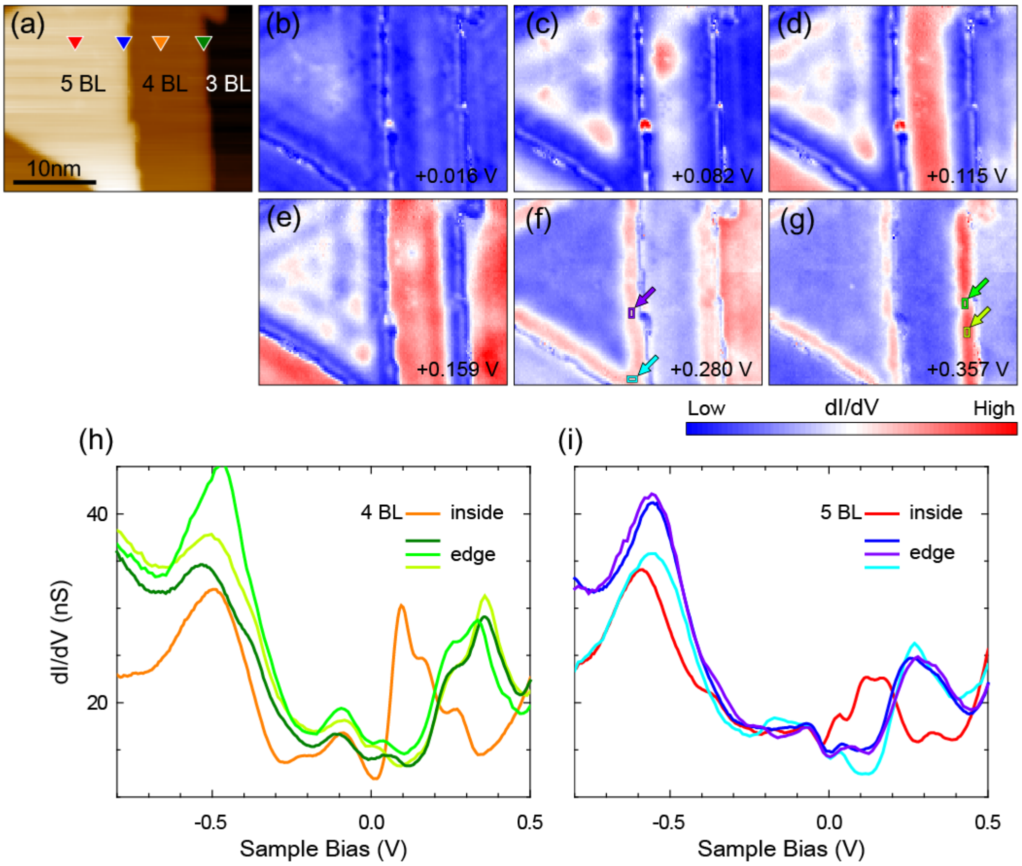

Supplementary Figure 2. STS maps for several energies near Sb 4 and 5 BL edge. (a) STM topographic image on a Sb island obtained simultaneously with the STS (*dI/dV*) maps. The *dI/dV* LDOS maps at several energies at (b) +0.016, (c) +0.082, (d) +0.115, (e) +0.0159, (f) +0.280, and (g) +0.357 eV. (h) and (i) *dI/dV* curves obtained along the edge channels but on defect sites indicated with colored boxes and arrows in (f) and (g) as compared with the *dI/dV* curves of Fig. 2e and f.

The STS (*dI/dV*) map of a Sb 4 BL film shows that the edge state distributes within about 2 nm from the edge. This matches well with the charge density plot of the edge states calculated for a Sb 4 BL nanoribbon.

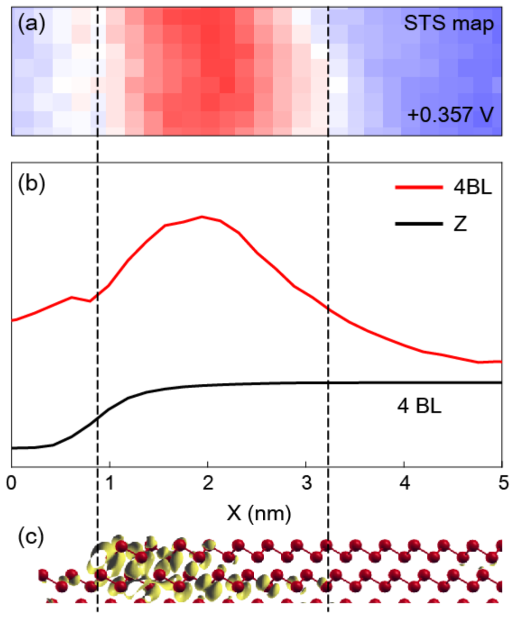

Supplementary Figure 3. Spatial distribution of Sb 4 BL's edge states (a) STS (*dI/dV*) map near the Sb 4 BL edge at +0.357 eV, which is taken from Fig. 2(b). (b) The averaged LDOS profile (red) of (a) together with the topographic line profile (black). (c) A charge density plot of the calculated edge states for a Sb 4 BL nanoribbon (the yellow blobs), which corresponds to the state indicated by the arrow in Fig. 4(g).

The quasiparticle interference is observed near the Sb islands edges [Supplementary Fig. 2]. This explains the apparent energy shift of the state at +0.1 eV to about 0.25 eV toward the edge of 4 BL [middle of (a) and (c)]. Since the other states [black dashed line in (c)] have no such dispersion, this cannot be due to a band bending effect. The distinction between this dispersing state and the edge state [green triangles in (c)] is unambiguous.

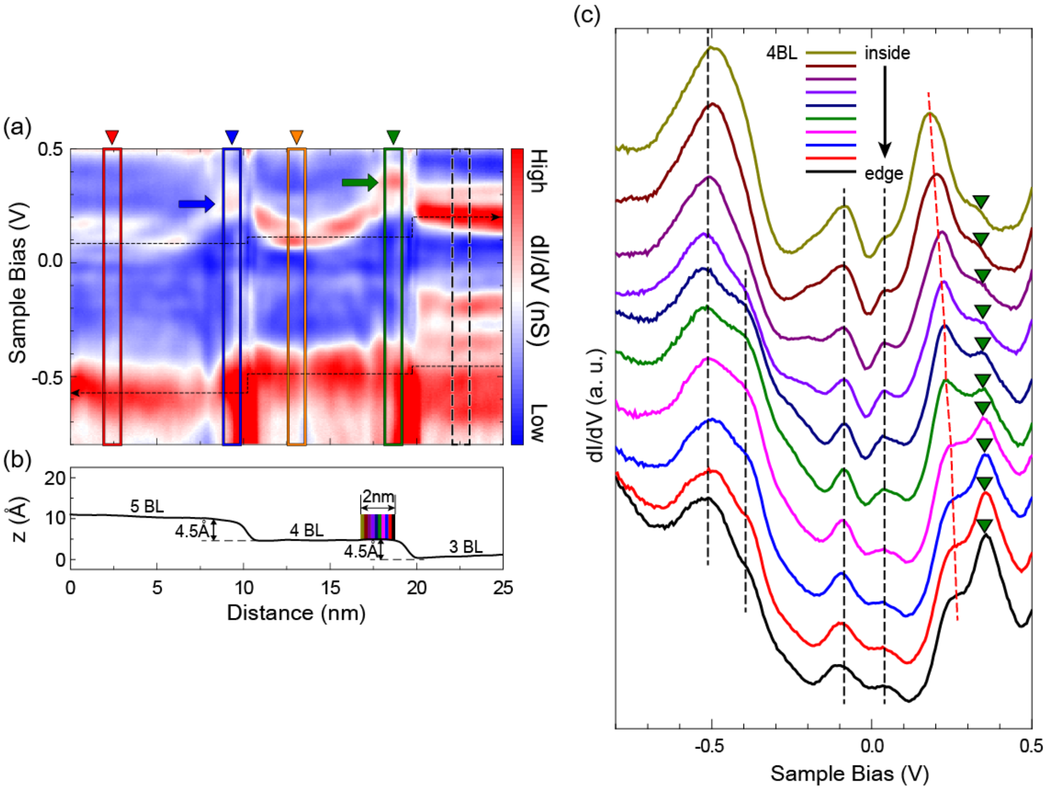

Supplementary Figure 4. The STS measurements near the Sb 4 BL edge. (a) The STS map crossing two step edges of 5 and 4 BL films, which is taken from Fig. 2d. (b) The topographic profile obtained during the STS measurements. (c) STS spectra near the Sb 4 BL edge. The line colors indicate the position where the STS obtained, which is indicated at (b). The energies of spectral features (the dashed lines) do not change except for one peak [the red dashed line], which is due the quasiparticle interference dispersion [Supplementary Fig. 2(b)-(e)].

Ab initio calculations for the edge states of Sb nanoribbon structures without and with the substrate. As shown in (a) and (b) the top layers have the step edge structures, which are fully relaxed. The trivial edge state with the Rashba spin splitting [2 BL case in (c)] and the non-trivial edge state with the Dirac dispersions [4 BL in (d)] are contrasted by their band dispersion near $\bar{\Gamma}$: In the case of 2 BL, the edge states converge into conduction bands. In contrast, the branches of the edge states of the 4 BL are separated into the conduction and the valance bands. If the spin-orbit coupling of the top layer of 4 BL is set to zero, the dispersion of the edge states become trivial merging into the conduction bands [(e)]. This difference is due to the topological phase transition and the band inversion occurring between 3 and 4 BL thickness. This nature is preserved even if the effect of the substrate is included [(f) and (g)].

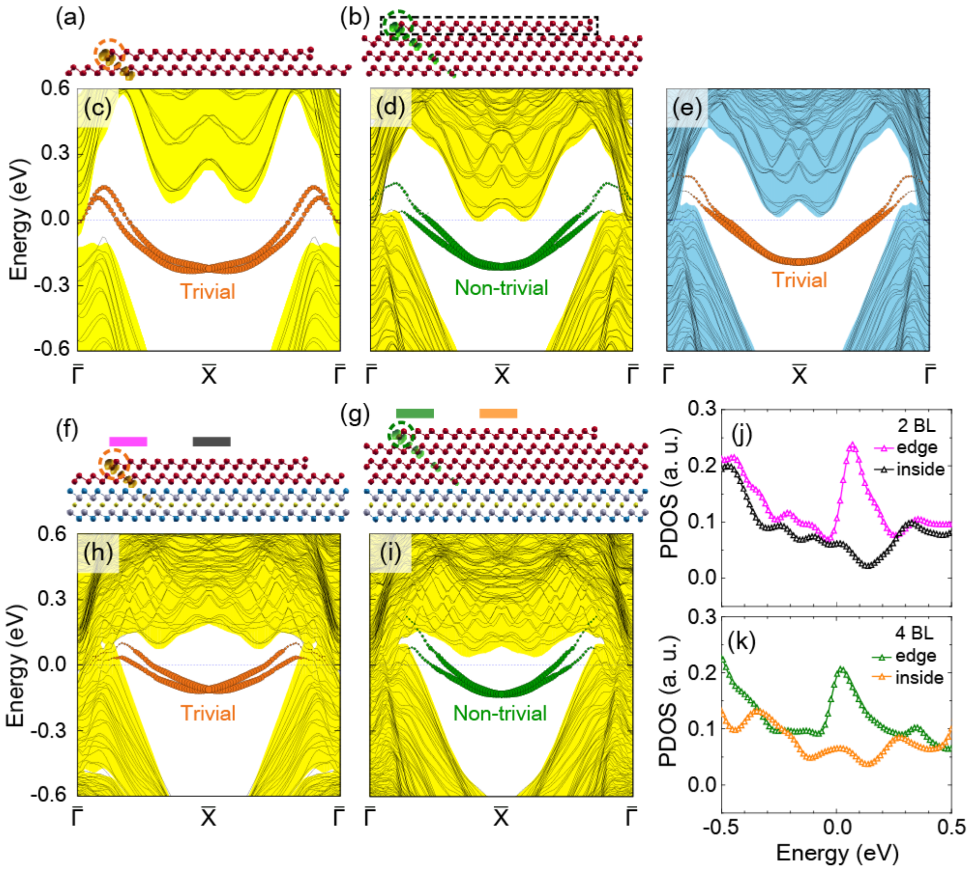

Supplementary Figure 5. The topological nature of the edge states for each Sb nanoribbon. Atomic structures used in the calculations for (a) Sb(111) 2 BL and (b) Sb(111) 4 BL in the floating geometry with zigzag step edges on the top layers. Calculated band structures of each model are depicted on (c) and (d) along the $\bar{\Gamma}-\bar{X}-\bar{\Gamma}$ direction. The bands represented by blue and green dots originate from step edges [the atoms within dashed circles in (a) and (b)], respectively, whose charge densities at $\bar{X}$ point are overlaid in the structure models. (e) The calculated band structure of the 4 BL nanoribbon with the spin-orbit coupling of the top layer [black dashed line in (b)] set to zero. (f) and (g) are structures for Sb(111) 2 BL and 4 BL films on Bi_2Te_2Se. The corresponding band structures are depicted in (h) and (i). (j) and (k) are the calculated LDOS of 2 BL and 4 BL film, which obtained from the colored boxes in (f) and (g).

When the Sb 4 BL film is grown on Bi_2Te_2Se, the potential gradient is generated because of the presence of the substrate. Also the broken inversion symmetry causes bands to split. Nevertheless, *ab initio* calculations verify that the QSH phase is preserved. This can be seen by the evolution of the calculated band structures with the distance between the Sb film and Bi_2Te_2Se varied to tune the substrate effect [(g)-(j)]. When the distance is set to the equilibrium position [(j)] with the full strength of the substrate effect, the band inversion and a non-trivial band gap are maintained. This substrate effect can be well understood by the electric field effect [(a)-(d)]. When the electric field is applied gradually, the bands split with the band inversion maintained. The band structure at 40 meV/Å is fully consistent with that with the substrate in (j) except for the rigid energy shift due to the doping. These results clearly prove that Sb 4 BL/Bi_2Te_2Se is a QSH system.

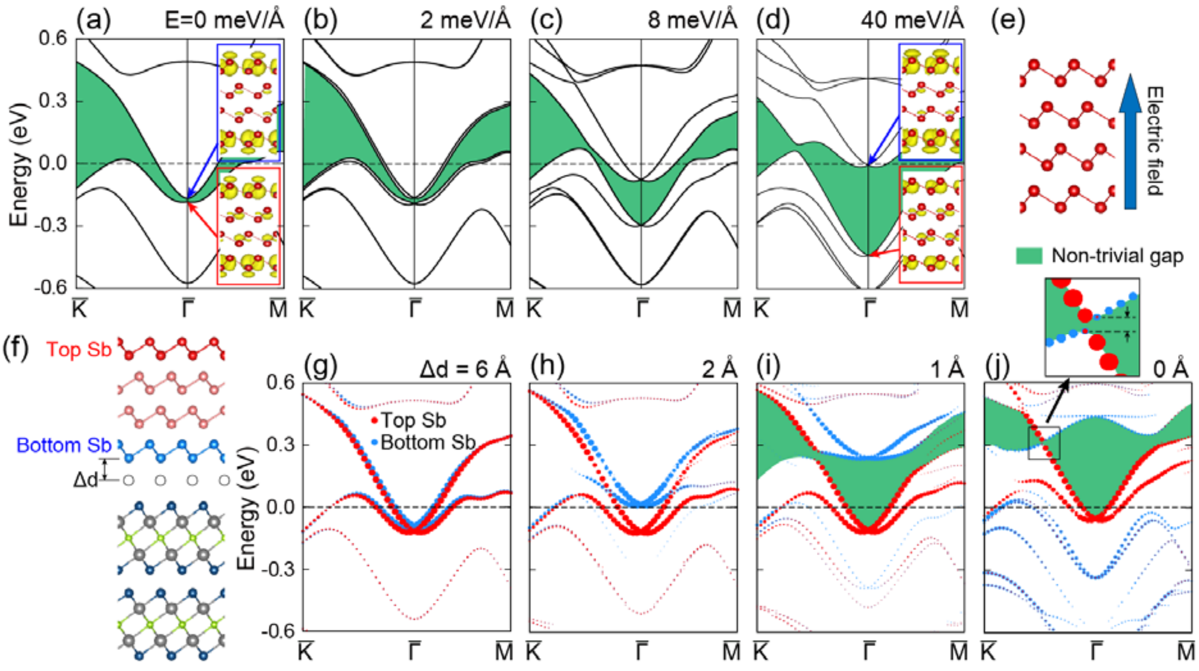

Supplementary Figure 6. *Ab* initio calculation for Sb 4 BL when the potential gradient or the substrate exists. (a)-(d) The calculated band structures when the electric field is applied with a strength of 0, 2, 8, and 40 meV/Å for a Sb 4 BL film as shown in (e). (f) Atomic structure of a Sb 4 BL on top of Bi_2Te_2Se. (g)-(j) Calculated band structures with the substrate at a distance of 6, 2, 1, and 0 Å from the equilibrium position, respectively. The inset of (j) enlarges the non-trivial band gap of Sb 4 BL/Bi_2Te_2Se.
